# Assessment of a Bioengineering and Nursing Student Partnership for Medical Product Design

**DOI:** 10.1007/s43683-025-00180-y

**Published:** 2025-03-24

**Authors:** Renee M. Clark, April A. Dukes, Lucille Sowko, Mark Gartner

**Affiliations:** 1 Department of Industrial Engineering, University of Pittsburgh, B-12 Benedum Hall, Pittsburgh, PA 15261 USA; 2 Engineering Education Research Center, University of Pittsburgh, Pittsburgh, PA USA; 3 School of Nursing, University of Pittsburgh, Pittsburgh, PA USA; 4 Department of Bioengineering, University of Pittsburgh, Pittsburgh, PA USA

**Keywords:** Bioengineering, Biomedical engineering, Nursing, Interprofessional, Product design, Partnership, Self-efficacy

## Abstract

**Purpose:**

In 2018, the Department of Bioengineering and the School of Nursing at University of Pittsburgh implemented an interdisciplinary partnership that integrated senior nursing students into the bioengineering capstone Senior Design course as part of a National Institutes of Health education grant. This two-semester course requires senior Bioengineering students to synthesize and extend principles from prior coursework toward the design a medical product meeting an unmet clinical need. Senior Design teams interact with clinicians, patients, and caregivers as part of the overall design process to understand the unique challenges of medical product design, including the requirements for regulatory approval. The teams develop iterative designs, fabricate prototypes, and perform both verification and validation testing to evaluate whether product performance criteria are met. Integrating nursing and bioengineering students was anticipated to provide opportunities for interprofessional learning, earlier and more frequent clinical input to the design process, and exposure to a spectrum of unmet clinical needs. Conversely, nursing students were anticipated to gain an understanding of the medical product design process, including regulatory requirements, to potentially empower future innovativeness.

**Methods:**

The impact of this interdisciplinary partnership on the anticipated outcomes was assessed over a five-year timeframe using research surveys and student interviews. The design self-efficacy survey was administered in a pre-post manner to assess changes in bioengineering and nursing students’ confidence, motivation, success expectancy, and apprehension for performing design activity. Students’ interprofessional collaborative development was also measured in a retrospective pre-post manner using the interprofessional collaborative competency attainment survey. Finally, a spectrum of student interviews was conducted to obtain perspectives about the interdisciplinary partnership. The data were analyzed using statistical and qualitative data methods.

**Results:**

The results were overwhelmingly positive for the partnership. The results make a strong case for such partnerships and suggest benefits for both student groups, including significant effects for design confidence and a multitude of collaborative competencies. For bioengineering students, the nursing students’ clinical knowledge, perspectives, suggestions related to unmet clinical needs, and feedback were mentioned by 84% of interviewees as a partnership benefit. The nursing students cited interprofessional teamwork as the most valuable benefit (71% of interviewees) and indicated that it supported their ability to be innovative.

**Conclusions:**

The results make a strong case for engineering and nursing schools to pursue and establish partnerships between their students. This study is situated in the literature as part of an evolving trend of partnership between these two professions.

## Introduction

Practical experiences are a key component of both the engineering and nursing undergraduate curriculums in the form of capstone design projects and clinical practicums, respectively. For bioengineering students who design a medical device as part of their capstone projects, they must ideally understand the context in which the device is used [1]. However, undergraduate bioengineering students typically have limited experience within clinical settings [1, 2]. Yet, being able to observe how users in a clinical environment interact with a device contributes to enlightened design activity [1]. Also, being able to directly communicate and collaborate with clinicians and patients supports the bioengineering student in realizing true unmet needs and ways in which they can innovate to solve these challenges [1]. Conversely, the nursing profession is becoming increasingly complex based (in part) on the use of new technologies within nursing practice as well as higher expectations to participate in the research and design of new medical devices [3–5].

Therefore, to better incorporate clinical perspectives and needs throughout the design process and empower both bioengineering and nursing students to make innovative design contributions to the healthcare industry, the Bioengineering Department and the School of Nursing at University of Pittsburgh partnered in 2018 to provide an interprofessional educational experience in which senior-level nursing students were embedded within bioengineering capstone design teams as part of their coursework.

The anticipated outcomes for this partnership included providing bioengineering students with a clinical resource to support the product design effort and facilitate their work in the clinic. It was anticipated that nursing students would learn the fundamentals of the product development process and participate in medical product design, prototyping, and validation testing. This initiative aligns with the call by the World Health Organization (WHO) for action on interprofessional education to better educate and prepare health care workers for collaborative practice to ultimately improve health outcomes [6]. The WHO defined interprofessional education as occurring when “students from two or more professions learn about, from and with each other to enable effective collaboration and improve health outcomes.” [6, p. 7].

To evaluate these envisioned outcomes, the interdisciplinary partnership was assessed over a five-year period via interviews and research surveys. The survey instruments were administered in a pre- and post-experience fashion to assess the degree of change in bioengineering and nursing students’ confidence, motivation, success expectancy, and apprehension in design activity as well as their interprofessional collaborative competency attainment. Team interviews were also conducted to gather students’ perspectives on the pairing of the two groups of students in a design setting. Our research questions were as follows:

1. When developing a medical product, what effect on design self-efficacy is associated with an engineering-nursing student collaboration?
2. When developing a medical product, what effect on interprofessional collaborative competencies attainment is associated with an engineering-nursing student collaboration?
3. When developing a medical product, what are students’ perspectives on an engineering-nursing student collaboration?

This article first reviews other published partnerships between nurses and engineering teams to position this study as part of an evolving trend of similar partnerships. The article then describes the two-semester capstone course in the Department of Bioengineering at University of Pittsburgh, the student participants, and the assessment methods. Finally, the assessment results from the research surveys and interviews are presented to demonstrate and describe the value of such partnerships.

## Literature Review

The recent literature contains numerous instances of interprofessional collaborations between the clinical and engineering domains to achieve outcomes including innovative healthcare solutions, infusion of clinical perspectives during design, and improved patient care. For example, the nursing and bioengineering domains have been officially “joined” by dual degree programs. The first dual degree program in the U.S. was offered at Duquesne University in Pittsburgh, where students earn a Bachelor of Science in Nursing and a Bachelor of Science in Biomedical Engineering as part of a five-year program [3]. In this program, student teams at Duquesne complete a two-semester capstone course in the 5^th^ year to produce an engineering solution to a healthcare problem based on their clinical experience, and the solution is tested via a prototype. Nurse-engineer graduates from this program are expected to have enhanced abilities to solve technically based clinical problems.

At Widener University, a series of three biomedical engineering courses was implemented to drive greater continuity and professional practice for engineering students [2]. The first two courses consisted of Biomechanics and Medical Devices, with the latter involving the pairing of engineering and nursing students to identify unmet needs in a real-world clinical setting. The final course consisted of a two-semester capstone project in which the engineering students executed their proposed solutions to the clinical challenge, which they had self-identified during one of the two previous courses. In a survey given at the end of the capstone course, all engineering students who responded agreed that they felt more confident in their understanding of the problem through the clinical immersion, believed they successfully met the unmet clinical need, and were encouraged as entrepreneurs. Clinical immersion programs provide another opportunity to enhance bioengineering students’ abilities to develop clinical solutions that are user centered. For example, at the University of Illinois at Chicago, the Clinical Immersion Program was initially offered as a summer internship for rising senior bioengineering students [1]. It offered six weeks of workshops and clinical rotations with an emphasis on developing needs statements. Medical students were introduced for interdisciplinary teamwork as well. In addition to collaborations with bioengineers, nurses have also collaborated with software developers to produce user-centered designs for digital health solutions [4].

In short, the literature contains examples of and calls for interdisciplinary collaboration among nurses and engineers to drive innovation, including during the early stages of solving healthcare problems [3, 5, 7]. In Zhou’s review of the literature, the prominent roles of the nurse in their interdisciplinary collaboration with engineers were as a (1) requirements analyst (i.e., identifying needs based on clinical experience and building context), (2) designer, (3) tester of the feasibility, usability, and acceptability of the design in a real-world setting, and (4) evaluator (i.e., checking whether healthcare results were ultimately achieved) [5].

As an extension to the literature, the present article contributes a formal mixed-methods assessment of an interdisciplinary, medical product design experience between undergraduate bioengineering and nursing students. Specifically, this study incorporates a structured analysis of student interviews as well as validated instruments from the literature to measure before versus after perceptions of interprofessional collaborative competency and design self-efficacy, including confidence, motivation, success expectancy, and apprehension.

## Methods

### Coursework

The capstone design course (Senior Design) in the Department of Bioengineering at University of Pittsburgh is a two-semester course that challenges teams of engineering students to design or redesign a medical product using a process aligned with the regulatory requirements in the United States and in the European Union (EU). More specifically, in the United States, these requirements correspond to the Food and Drug Administration’s Quality System Regulation 21 CFR 820 [8]. In the European Union, these requirements correspond to Regulation (EU) 2017/745 [9]. Bioengineering teams must go “outside the classroom” to conduct ethnographic activities with patients, clinicians, and caregivers. Beginning in 2018, senior nursing students in their Transitions to Professional Practice course (“Transitions”) were offered an opportunity to be embedded within bioengineering capstone design teams as an interdisciplinary initiative by the Department of Bioengineering and School of Nursing. Each interprofessional team is often composed of one (or two) nursing student(s) and five bioengineering students. The nursing students are awarded clinic hours for participating on a capstone design team if enrolled in the Transitions course. However, they may also participate by way of a one credit Independent Study course. Senior nursing students are ideal participants in Senior Design teams since they have completed six of nine clinical practicums by the time they reach senior status. Nursing students may participate on a design team during the fall and/or spring semesters. The nursing and bioengineering students are paired using a self-selection process after the capstone instructor assists with some initial matching based on the nursing students’ interests.

Senior Design teams focus the first six weeks of the fall semester on ethnography activities as the basis of identifying and objectively evaluating unmet clinical needs, which serve as the basis for their capstone design project topic. Clinic visits by the team for ethnographic studies are coordinated through the student nurse and the nursing professor. During the fall semester, the team formulates user needs, use cases, risk analyses, and a quantitative design specification to guide prototype development. Teams also begin planning verification and validation testing activities that will be conducted in the spring semester. All supporting documents are contained in a design history file that each team prepares and maintains. In the spring semester, the team focuses on design iterations, design freeze, and formal verification and validation testing of the engineering prototype.

Nursing students provide rapid feedback to guide requirements formulation, prototype evaluation, and verification and validation (V&V) test planning. Design teams may work with the University of Pittsburgh Institutional Review Board (IRB) depending on whether V&V testing includes human subjects’ research. To provide context on the nature of these two-semester projects, below are several past project titles:

- Detection and alert system for neonatal nasal cannula dislodgement
- Head support for amyotrophic lateral sclerosis (ALS) patients
- Sharps disposal in under-resourced communities
- Intravenous stabilization at elbow joint
- Pediatric intravenous site stabilization device
- Automated syringe drug push
- Robotic toothbrush holder: assistive technology for hands-free teeth brushing
- Improved foley bag
- Avoiding air bubbles in intravenous tubing
- Nasogastric tube securement

### Data Collection

Student survey and interview data were collected starting in the fall 2018 semester when nursing students were first embedded in bioengineering design teams. Data were collected until the spring 2024 semester, excluding the 2020–2021 academic year due to the COVID-19 pandemic. This resulted in five years of student data for this research study. The University of Pittsburgh Institutional Review Board granted approval for this study (PRO18080267 and STUDY20120194). During this five-year timeframe, 408 bioengineering students and 95 nursing students were part of 72 design teams.

### Assessment

Outcomes assessed in this study for the bioengineering and nursing students included (1) their design self-efficacy in a pre- and post-experience fashion, (2) their interprofessional collaborative competencies in a retrospective pre-post fashion, and (3) their perspectives on the interprofessional design team experience and partnership. Outcomes were assessed for the bioengineering and nursing students separately. Students entered self-selected study codes to the Qualtrics online surveys, so pre- and post-responses could be matched.

#### Design Self-Efficacy

This survey instrument by Adam Carberry and colleagues measures a person’s degree of confidence, motivation, success expectancy, and anxiety/apprehension in performing engineering design overall (ED) as well as eight (8) specific steps of the engineering design process (EDP) [10]. Thus, there are nine items associated with each of the above four outcomes, for a total of 36 items in this reliable, validated instrument. The respondent is asked to rate their degree of confidence, motivation, success expectancy, and anxiety/apprehension in performing specific design-related tasks on an 11-point ordinal scale ranging from 0 (low) to 100 (high). For engineering design overall (ED), the one task is “Conduct engineering design.” Relative to the engineering design process (EDP), the eight (8) design-related tasks are as follows:

- Identify a design need
- Research a design need
- Develop design solutions
- Select the best possible design
- Construct a prototype
- Evaluate and test a design
- Communicate a design
- Redesign

The scores of these eight task items are averaged to obtain the engineering design process (EDP) score for each outcome—confidence, motivation, success expectancy, anxiety/apprehension. We calculated values of Cronbach’s alpha, a measure of internal-consistency scale reliability, for the pre-responses as well as the post-responses [11]. An alpha value was calculated for each of the four outcomes using the eight (8) task items per outcome, as done by Carberry et al. during the instrument development process [10].

#### Interprofessional Collaborative Competency Attainment

This instrument, abbreviated as ICCAS, was developed by Colla MacDonald and colleagues and measures the degree of interprofessional collaborative practice [12–14]. It uses a retrospective pre-post design and asks respondents to simultaneously rate their abilities “before” and “after” participating in the interprofessional design team experience. The response scale is ordinal and ranges from 1 to 5, with 1 = poor to 5 = excellent. The following are sample abilities (i.e., items) that are concurrently rated by the respondent with respect to before and after the interprofessional design experience:

- Actively listen to interprofessional team members’ ideas and concerns.
- Work effectively with interprofessional team members to enhance care.
- Recognize how others’ skills and knowledge complement and overlap with my own.
- Take into account the ideas of interprofessional team members.

The instrument contains a final question on a 1–5 scale that asks respondents the following question: Compared to the time before the interprofessional design team experience, I would say my ability to collaborate interprofessionally is (1 = much worse now to 5 = much better now).

#### Statistical Analysis of Survey Data

Statistical analysis of the survey data was conducted using IBM SPSS 29.0. A paired-samples *t* test for dependent populations was conducted with the data from both the Design Self-Efficacy survey (pre- vs. post-experience) and the ICCAS (retrospective pre- vs. post-experience). From the Design Self-Efficacy survey, the various ED and EDP differences were approximately normally distributed for the bioengineering students. For the nursing students, for whom the sample size was smaller, the distribution of the differences could not be determined with certainty given the smaller *n*. Therefore, the non-parametric equivalent, the Wilcoxon matched-pairs signed-rank test, was run for the nursing students’ self-efficacy data to corroborate the results from the paired-samples *t* test, with both sets of results displayed for the reader [11].

For the ICCAS data for both the bioengineering and nursing students, the differences were not normally distributed based on a visual examination. Although the sample sizes were not necessarily small, the non-parametric Wilcoxon matched-pairs signed-rank test was also run to corroborate the *t* test results for conservativeness.

Practical significance of the differences was assessed using Cohen’s *d* effect size or Hedges' *g* effect size, with the latter used when the sample size is small [15]. An effect size of 0.20 is considered small, 0.50 is considered medium, and 0.80 or higher is considered large [16]. Because of the multiple survey items (and therefore statistical tests run), Bonferroni’s correction for multiple comparisons was considered [17, 18]. The final question on the ICCAS was analyzed using a z-test of proportions and an odds ratio for practical significance, with an odds ratio of greater than 3 considered large [19].

#### Team-Based Interviews

Semi-structured, team-based interviews were conducted during class time, initial networking events for the bioengineering and nursing students, and end-of-term design expos. These interviews were conducted by two members of the research team, and the questions posed are given in Table 1.

**Table 1:** Interview questions

Interview question	Interviewee student group	Cohen’s kappa κ
1. What are your thoughts and impressions of embedding a nursing student in your design team, including benefits or drawbacks?	Bioengineering	0.86
2. How (if at all) has an embedded nursing student helped you in discovering unmet customer needs?	Bioengineering	0.80
3. Has embedding a nursing student in your design team assisted in receiving quick feedback from clinical customers or stakeholders?	Bioengineering	0.85
4. What are your thoughts and impressions of being embedded on an interprofessional design team including benefits or drawbacks?	Nursing	0.72
5. Do you believe you can be more innovative in your clinical practice in both recognizing and developing solutions for unmet clinical needs?	Nursing	0.64
6. What skills or knowledge did you gain from the bioengineering students that were part of the design team?	Nursing	Not coded

A content analysis of the interview responses was conducted by two research analysts to determine the frequent themes and categories discussed by the students [20]. Coding schemes were developed by the first author using an emergent, data-led approach that entailed initially reading all responses to identify the relevant, recurrent, and/or important themes [21]. During the content analysis, the two analysts independently coded the responses using the coding schemes. The analysts then met to discuss each code assigned to ensure consensus. Thus, all responses were double-coded. The first-time inter-rater reliability scores for the two analysts based on Cohen’s kappa (κ) are given in Table 1. These Kappa (κ) scores range from 0.64 to 0.86, indicating fair to strong agreement beyond chance [11]. The coding schemes are shown in the Results section with the outcomes.

## Results

We collected matched (i.e., pre and post) survey responses from 90 bioengineering and 20 nursing students. Among the bioengineering respondents, approximately 37% identified as male, 55% identified as female, and 8% indicated another gender or did not provide a response. Similarly, among the nursing respondents, about 20% identified as male, 75% identified as female, and 5% either identified as another gender or did not provide a response.

### Design Self-Efficacy

#### Bioengineering Students

The largest effect associated with the interprofessional design experience for the bioengineering students occurred for their confidence in engineering design (ED) overall as well as for the 8-step engineering design process (EDP), with Cohen’s *d* = 0.70 for each, as shown in Table 2. The pre-to-post confidence differences were also significantly different from zero, with *p* < 0.001 for both ED and EDP. These differences remained statistically significant even after application of the highly conservative Bonferroni correction for multiple comparisons.

**Table 2:** Bioengineering students’ design self-efficacy results (0 = low to 100 = high**)**

	Mean (std dev) pre	Mean (std dev) post	*n*	*p*	Effect Size *d*
Confidence in ED	73.96 (16.59)	84.51 (12.04)	91	**< 0.001**	0.70
Confidence in EDP	77.08 (12.57)	85.07 (9.68)	92	**< 0.001**	0.70
Motivation for ED	79.23 (18.15)	79.23 (17.40)	91	1.000	0.00
Motivation for EDP	80.45 (12.49)	79.45 (15.10)	91	0.448	-0.08
Success in ED	74.49 (13.98)	80.45 (12.33)	89	**< 0.001**	0.42
Success in EDP	78.06 (10.41)	81.81 (10.44)	89	**< 0.001**	0.40
Anxiety with ED	40.34 (26.28)	33.18 (25.49)	88	0.027	-0.24
Anxiety with EDP	36.25 (21.38)	33.20 (23.51)	88	0.290	-0.11

Statistically significant results at *α* = 0.05 after Bonferroni correction are in bold.

The second largest effect for the bioengineering students occurred for their expectancy of success, with *d* = 0.42 (*p* < 0.001) for ED overall and *d* = 0.40 (*p* < 0.001) for the engineering design process steps (EDP). For bioengineering students, there was a small desirable effect relative to their anxiety/apprehension with engineering design ED (*d* = − 0.24; *p* = 0.027) and the design process (*d* = − 0.11; *p* = 0.290) associated with the interprofessional experience. However, for the bioengineering students, there was not an effect for their design-related motivation associated with the interprofessional experience, as shown in Table 2 (*d* = 0.00 and *d* = − 0.08 for ED and EDP, respectively).

#### Nursing Students

Like the bioengineering students, the largest effect associated with the interprofessional design experience for the nursing students occurred relative to their confidence in engineering design (ED) overall and the 8-step engineering design process (EDP), with Hedge’s *g* = 1.19 and *g* = 0.71, respectively, as shown in Table 3. Note the relatively low level of overall engineering design (ED) confidence at the start of the experience for the nursing students (M = 29.00) and the associated large effect of *g* = 1.19. Also note that the effect sizes for confidence were larger for the nursing students than for the bioengineering students.

**Table 3:** Nursing students’ design self-efficacy results (0 = low to 100 = high)

	Mean (std dev) pre	Mean (std dev) post	*n*	*p*	Non-parametric *p*	Effect size *g*
Confidence in ED	29.00 (21.00)	58.50 (20.84)	20	**<0.001**	**<0.001**	1.19
Confidence in EDP	59.06 (18.46)	74.13 (15.91)	20	**0.004**	**0.002**	0.71
Motivation for ED	57.00 (29.75)	66.50 (19.81)	20	0.106	0.117	0.36
Motivation for EDP	71.88 (24.37)	77.63 (17.22)	20	0.317	0.867	0.22
Success in ED	46.00 (25.21)	52.00 (25.26)	20	0.272	0.270	0.24
Success in EDP	63.13 (17.95)	70.94 (15.12)	20	0.105	0.076	0.37
Anxiety with ED	63.00 (26.58)	56.50 (28.70)	20	0.318	0.241	-0.22
Anxiety with EDP	41.13 (21.44)	41.31 (23.57)	20	0.978	0.925	0.01

Statistically significant results at *α* = 0.05 after Bonferroni correction are in bold.

The pre-to-post differences in confidence were significantly different from zero, with *p* < 0.001 for ED. For the EDP, *p* = 0.004 and *p* = 0.002 for the parametric and non-parametric tests, respectively. These differences remained statistically significant after application of the Bonferroni correction. There were small, non-significant effects for the nursing students’ motivation and success expectancy for ED and the EDP, with effect sizes ranging from *g* = 0.22 to *g* = 0.37 in Table 3. This differed from the bioengineering students, for whom there was no effect for design-related motivation associated with the experience. Consistent with the bioengineering students, however, there was a small desirable effect for design-related anxiety/apprehension for the nursing students, with *g* = − 0.22 for engineering design (ED) overall.

We calculated values of Cronbach’s alpha in the range of *α* = 0.89 to *α* = 0.93 for all of the pre-responses from the Design Self-Efficacy instrument and values of *α* = 0.90 to *α* = 0.96 for all of the post-responses from the instrument. These ranges indicated strong internal reliability [11].

### Interprofessional Collaborative Competency

#### Bioengineering Students Before and After

As shown in Table 4, the effect sizes for the various collaborative abilities as part of an interprofessional (IP) team were of medium magnitude or larger. They ranged from *d* = 0.53 (ability to address team conflict) to *d* = 0.99 (ability to use an IP team approach with the patient to assess the health situation) for the bioengineering students when they compared their abilities “after” versus “before” the interprofessional experience. All differences were significantly different from zero based on both the paired-samples *t* test and Wilcoxon matched-pairs signed-rank test (*p* < 0.001), and these differences remained significant after application of the Bonferroni correction.

**Table 4:** Bioengineering students’ interprofessional collaborative competency results

Ability (1 = poor to 5 = excellent)	Mean (std dev) before	Mean (std dev) after	*n*	*p*	Effect size *d*
Promote effective communication among members of an interprofessional (IP) team	3.75 (0.88)	4.40 (0.58)	106	<0.001	0.83
Actively listen to IP team members’ ideas and concerns	4.08 (0.85)	4.51 (0.56)	106	<0.001	0.62
Express my ideas and concerns without being judgmental	3.92 (0.87)	4.40 (0.63)	106	<0.001	0.64
Provide constructive feedback to IP team members	3.68 (0.85)	4.23 (0.68)	106	<0.001	0.81
Express my ideas and concerns in a clear, concise manner	3.80 (0.85)	4.34 (0.71)	105	<0.001	0.78
Seek out IP team members to address issues	3.61 (0.88)	4.18 (0.78)	106	<0.001	0.78
Work effectively with IP team members to enhance care	3.79 (0.87)	4.37 (0.71)	105	<0.001	0.86
Learn with, from and about IP team members to enhance care	3.47 (0.94)	4.19 (0.75)	105	<0.001	0.92
Identify and describe my abilities and contributions to the IP team	3.57 (0.90)	4.29 (0.69)	106	<0.001	0.91
Be accountable for my contributions to the IP team	4.06 (0.87)	4.52 (0.57)	106	<0.001	0.64
Understand the abilities and contributions of IP team members	3.80 (0.86)	4.37 (0.64)	106	<0.001	0.69
Recognize how others’ skills and knowledge complement and overlap with my own	3.61 (0.92)	4.41 (0.61)	106	<0.001	0.98
Use an IP team approach with the patient to assess the health situation	3.36 (1.03)	4.20 (0.75)	106	<0.001	0.99
Use an IP team approach with the patient to provide whole person care	3.08 (1.06)	3.91 (0.90)	106	<0.001	0.95
Include the patient/family in decision-making	2.93 (1.12)	3.55 (1.10)	106	<0.001	0.68
Actively listen to the perspectives of IP team members	3.77 (0.84)	4.40 (0.60)	106	<0.001	0.80
Take into account the ideas of IP team members	3.92 (0.75)	4.41 (0.60)	106	<0.001	0.68
Address team conflict in a respectful manner	3.90 (0.88)	4.34 (0.72)	106	<0.001	0.53
Develop an effective care plan with IP team members	3.36 (1.01)	3.93 (0.99)	105	<0.001	0.74
Negotiate responsibilities within overlapping scopes of practice	3.54 (0.92)	4.12 (0.90)	106	<0.001	0.79

The second and third-largest effects for the bioengineering students were *d* = 0.98 (ability to recognize how others’ skills and knowledge complement and overlap with my own) and *d* = 0.95 (ability to use an IP team approach with the patient to provide whole person care), respectively. These three largest effects highlight the impact on the bioengineering students with regard to their opportunity to interface with their end users (e.g., patients), which they otherwise might not be able to do, as well as their realization of the importance of the nurses’ contributions to the design effort.

### Nursing Students Before and After

As shown in Table 5, all but one effect size for the various collaborative abilities were large for the nursing students. These large effects ranged from *d* = 0.82 to *d* = 1.53, with the latter corresponding to the ability to understand the abilities and contributions of IP team members. All differences were significantly different from zero based on both the paired-samples *t* test and Wilcoxon matched-pairs signed-rank test (*p* < 0.001), and these differences remained significant after application of the Bonferroni correction. The second and third-largest effects for the nursing students were *d* = 1.32 (ability to provide constructive feedback to IP team members) and *d* = 1.30 (ability to seek out IP team members to address issues), respectively. These three largest effects highlight the impact on the nursing students with regard to their abilities to create direct interactions with the bioengineering students and understand what engineers can offer.

**Table 5:** Nursing students’ interprofessional collaborative competency results

Ability (1 = poor to 5 = excellent)	Mean (std dev) before	Mean (std dev) after	*n*	*p*	Effect size *d*
Promote effective communication among members of an interprofessional (IP) team	3.19 (0.93)	4.06 (0.62)	32	<0.001	1.24
Actively listen to IP team members’ ideas and concerns	3.75 (0.88)	4.47 (0.57)	32	<0.001	0.99
Express my ideas and concerns without being judgmental	3.29 (1.13)	4.16 (0.69)	31	<0.001	1.03
Provide constructive feedback to IP team members	3.00 (0.79)	4.13 (0.78)	30	<0.001	1.32
Express my ideas and concerns in a clear, concise manner	3.13 (0.92)	4.06 (0.57)	31	<0.001	1.15
Seek out IP team members to address issues	2.94 (0.89)	3.97 (0.80)	31	<0.001	1.30
Work effectively with IP team members to enhance care	3.32 (1.08)	4.32 (0.70)	31	<0.001	1.04
Learn with, from and about IP team members to enhance care	3.22 (0.94)	4.13 (0.71)	32	<0.001	1.06
Identify and describe my abilities and contributions to the IP team	3.00 (0.97)	4.12 (0.74)	33	<0.001	1.21
Be accountable for my contributions to the IP team	3.24 (0.94)	4.12 (0.70)	33	<0.001	0.98
Understand the abilities and contributions of IP team members	3.03 (0.77)	4.18 (0.73)	33	<0.001	1.53
Recognize how others’ skills and knowledge complement and overlap with my own	3.27 (0.94)	4.21 (0.70)	33	<0.001	1.26
Use an IP team approach with the patient to assess the health situation	3.09 (1.01)	4.09 (0.77)	33	<0.001	1.03
Use an IP team approach with the patient to provide whole person care	3.09 (0.95)	3.94 (0.79)	33	<0.001	0.85
Include the patient/family in decision-making	3.73 (0.94)	4.24 (0.79)	33	<0.001	0.68
Actively listen to the perspectives of IP team members	3.61 (0.93)	4.27 (0.63)	33	<0.001	0.82
Take into account the ideas of IP team members	3.64 (0.90)	4.39 (0.61)	33	<0.001	1.07
Address team conflict in a respectful manner	3.45 (1.09)	4.09 (0.77)	33	<0.001	0.91
Develop an effective care plan with IP team members	3.16 (0.85)	4.00 (0.88)	32	<0.001	1.05
Negotiate responsibilities within overlapping scopes of practice	3.06 (0.90)	4.06 (0.79)	33	<0.001	1.16

The ICCAS results in Tables 4 and 5 indicate that the design experience may have been particularly impactful for the nursing students, given the 19 large effects for them and 9 for the bioengineering students. Nonetheless, these results indicate an impactful experience for both groups of students. For the nursing students, the smallest effect of *d* = 0.68 was associated with the ability to include the patient/family in decision-making. The small effect was due in part to the large “before” mean value of 3.73, which makes sense given the senior-level nursing students’ clinical exposure at that point in their educations.

We obtained Cronbach’s alpha values of *α* = 0.95 for all retrospective before responses and *α* = 0.93 for all retrospective after responses, indicating strong internal reliability [11].

### Overall Ability to Collaborate Interprofessionally

**Fig. 1:**
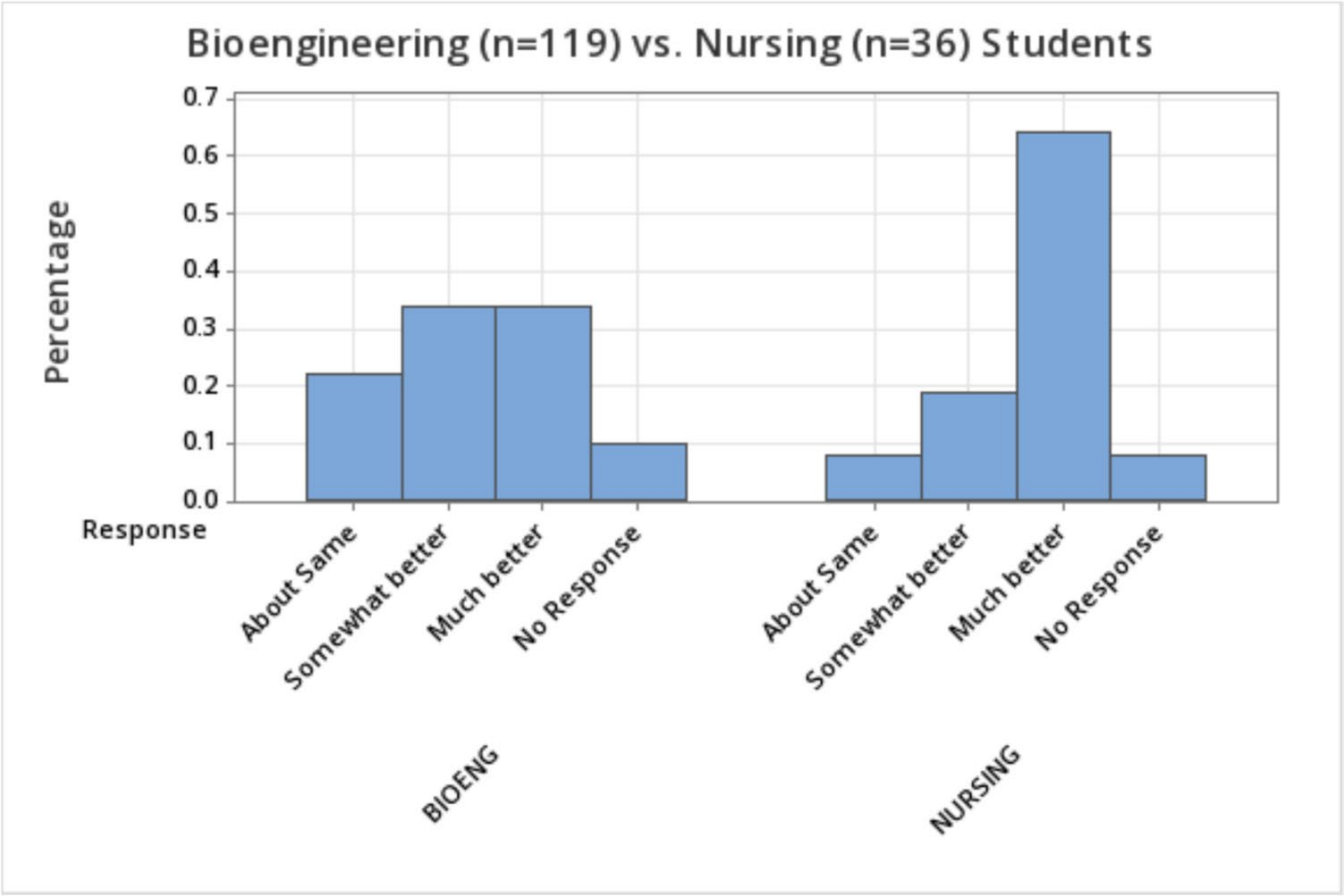
Ability to collaborate interprofessionally

Figure 1 presents the results to the question *Compared to the time before the interprofessional design team experience, I would say my ability to collaborate inter-professionally is…*

Figure 1 shows the much larger percentage of nursing students (64%) versus bioengineering students (34%) who reported a “much better” ability to collaborate interprofessionally after the design experience. These proportions were significantly different based on a z-test (*p* = 0.001) and had a large odds ratio of 3.49. In general, a higher percentage of nursing (versus bioengineering) students reported some degree of betterment in overall interprofessional collaboration, with 83% of nursing students reporting betterment versus 68% of bioengineering students.

Again, based on these results, the interprofessional design experience may have been particularly impactful for the nursing students, although most bioengineering students stated some degree of betterment in their interprofessional collaborative abilities.

### Interview and Content Analysis Results

#### Q1: Benefits & Drawbacks of Embedding Nursing Students

The bioengineering students overwhelmingly identified clinical experience, knowledge, and perspectives as a benefit to having a nursing student on their design teams, with 84% of bioengineering interviewees freely stating this. As described in Table 6, the nursing students served as consultants, had “inside” clinical knowledge, and were sources of feedback and unmet needs ideas. Also, 46% of interviewees cited the nurses’ access to clinical settings and clinical contacts, often leading to a greater ability to test prototypes. For example, these benefits are embodied in the following statement:

“The nurse has insight into patient care, what patients need, and the issues they face. She has a different perspective. Our nurse also had dental contacts.”

**Table 6:** Interview Q1 results from bioengineering students (κ = 0.86)

Q1. What are your thoughts and impressions of embedding a nursing student in your design team, including benefits or drawbacks?		
Category description	Benefit/drawback	% of interviewees (*n* = 101)
Nurses serve as consultant and have medical knowledge; nurses have inside knowledge of equipment, procedures, processes, patients, hospitals; nurses are source of ideas, unmet needs, new perspectives, and feedback.	Benefit	84%
Access to the clinical setting or equipment/materials; Nurses have clinical contacts; enhanced ability to test and implement.	Benefit	46%
Nurses have a “people” focus or orientation, including general communications skills.	Benefit	4%
Teamwork among different professions or disciplines.	Benefit	3%
Terminology/lingo difficulties, learning curves, communications issues, scheduling issues, team size, differences in opinions or disagreements, “fit” issues	Drawback	22%
Nurses’ lack of experience with design and solution generation/implementation.	Drawback	17%
Nurses’ time commitment and/or role are uncertain; may conflict with nurse’s other time commitments	Drawback	11%

Although the bioengineering students identified some drawbacks, such as team management and learning curve issues (22% of interviewees), 99% of the interview responses contained at least one benefit to embedding nursing students on the design team.

#### Q2: Nursing Student & Unmet Needs

In response to interview Q2 on how the nursing students helped with discovering unmet needs, the bioengineering students responded with various roles the nurses had on the team, as given in Table 7. These included a feedback source (42% of interviewees) and/or the source or provider of the chosen project (27%). For example, respectively,

“She has helped with narrowing them (needs) down, and what is helpful versus not, enabling the discarding of ideas that aren't good.”

“The nurse was the source of the unmet need. The nurse had worked on a cardio thoracic floor, where a patient inquired about the issue of taking his own blood pressure when he went home. This is how the unmet need was found.”

**Table 7:** Interview Q2 results from bioengineering students (κ = 0.80)

Q2. How (if at all) has an embedded nursing student helped you in discovering unmet customer needs?	
Category description	% of interviewees (*n* = 55)
Acknowledgment of the helpfulness/value of nurse’s clinical experience, knowledge, or perspective	64%
Nurse was source of feedback on project ideas, including narrowing or refinement of them	42%
Nurse was source or provider of chosen project	27%
Nurse enabled (or will enable) access to clinical settings, such as testing labs, hospitals, etc.	27%
Nurse enabled (or will enable) access to clinical contacts, mentors, resources, or end users	25%

In addition, the nursing student was an enabler of access to clinical spaces (27%) as well as clinicians or patients (25%). However, beyond specific roles held, the most frequent response (64%) was an acknowledgment of the value of the nurse’s clinical experience, knowledge, and perspective. For example,

“We have no clinical experience, and she has done two rotations in the hospital and is on the ground. This is beneficial, as we have no idea what is going on in that world.”

Interestingly, the most frequent explanation for discovering unmet needs (i.e., nurses’ clinical experience and knowledge) coincided with the most frequent benefit of embedding nursing students on the teams (Table 6).

#### Q3: Nursing Student & Quick Feedback

Bioengineering student responses to Q3 offered insight on how nursing students contributed to rapid feedback for the team. Specifically, the student nurses provided connections to various clinical contacts and obtained feedback for the team (55% of interviewees), as explained in Table 8. For example,

“She helped at the meetings and knowing the people to send emails to. She's also able to get quick email responses because she knows these people and has a personal connection with them, and thus it’s not just a random email being received by someone who doesn’t know us.”

**Table 8:** Interview Q3 results from bioengineering students (κ = 0.85)

Q3. Has embedding a nursing student in your design team assisted in receiving quick feedback from clinical customers or stakeholders?	
Category description	% of Interviewees (*n* = 40)
Nurse provides (or can provide) connection to clinical sponsor, mentor, resource, contact, or customer/end user, including obtaining feedback	55%
Nurse source or potential source of direct feedback on the design	43%
Nurse enabled access to clinical settings, such as testing labs and hospitals	3%

However, almost as prevalent, the nursing students themselves directly provided feedback to the design team (per 43% of interviewees). For example,

“This has been the most useful aspect. The nurses tell us how they will use the device and whether it needs to be more intuitive for the user. We want the nurses to help us determine how to best use the device.”

“The nurses will say ‘this sounds do-able or reasonable.’”

#### Q4: Benefits & Drawbacks of Being Embedded on a Design Team

Although the nursing students acknowledged the benefit of their clinical knowledge and perspective to being embedded group members (59% of interviewees in Table 9), the benefit they cited most frequently was the ability to work as part of a team and establish working relationships with the bioengineering students (71% of interviewees). A nursing student stated,

“It's great to work more with people impacting the medical field. It will give us a chance to interact with them. It's important to build relationships.”

**Table 9:** Interview Q4 results from nursing students (κ = 0.72)

Q4. What are your thoughts and impressions of being embedded on an interprofessional design team including benefits or drawbacks?		
Category description	Benefit/drawback	% of interviewees (*n* = 17)
Working as part of a group or team; establishing working relationships	Benefit	71%
Nurses serve as consultant and have medical knowledge; nurses have inside knowledge of equipment, procedures, processes, patients, hospitals; nurses are source of ideas, unmet needs, new perspectives, and feedback	Benefit	59%
Nurses become part of the solution or solution process, including gaining knowhow on how to do so	Benefit	47%
Access to the clinical setting; Nurses have clinical contacts; Enhanced ability to test and implement	Benefit	18%
Terminology/lingo difficulties, learning curves, communications issues, scheduling issues, team size, differences in opinions or disagreements	Drawback	12%
Nurses’ time commitment and/or role are uncertain	Drawback	6%

The nursing students were pleased with being a part of the solution and gaining knowhow on the solution process versus being just a “reporter” of problems (47% of interviewees). For example,

“I had had no prior exposure to engineering. I now have a better understanding of moving from an idea to something tangible, which is very cool. I want to fix issues that exist and that I have seen in healthcare, and I now understand how to go about solving patient issues.”

All interview responses from the nursing students contained at least one benefit to being embedded on the design team, with the frequency of perceived drawbacks being low as shown in Table 9.

#### Q5: Belief in Being More Innovative

Of the *n* = 16 nursing students who were asked about their ability to be more innovative in their clinical practice, nearly all (94%) believed they could be. This was due to items such as teamwork as well as working directly in the clinical setting, as shown in Table 10. For example,

“It definitely does. This type of interprofessional team experience will have a positive impact on the nursing profession. Nowadays, the nurse has more autonomy, and nurses are the ones mainly dealing with the technology. This experience will expand nurses’ views and experiences and introduce them to bioengineering. It will push them to explore further.”

“Yes. It has given me perspective on the process to get a new product to market. I can now envision collaborations with other nurses if such a team is available to me in the future. It has given me an ‘edge’ to being creative. It has boosted my confidence that a change can be made with an idea I have.

**Table 10:** Interview Q5 results from nursing students (κ = 0.64)

Q5. Do you believe you can be more innovative in your clinical practice in both recognizing and developing solutions for unmet clinical needs?	
Category description	% of interviewees (*n* = 16)
Interprofessional teamwork drives innovativeness	56%
Ideas occur to nurses just by virtue of working in the clinical setting	56%

#### Q6: Skills or Knowledge Gained from Bioengineering Students

This section contains a subset of responses to interview Q6 about the skills and knowledge nursing students gained from the bioengineering students on the design team. The responses demonstrated that nursing students gained an appreciation for or knowledge of engineered products, the engineering design process, basic sketching skills, and interprofessional teamwork and communication, as follows:

“Presenting a product and sharing a vision about it in a clear, concise way. (I presented in clinical). I also learned about engineering sketching in a workshop.”

“I learned to sketch in a workshop. I did not know how to sketch beforehand but came out with basic skills. I also learned about working on an inter-professional team and working with different types of professionals. I learned to put things in context when communicating.”

“Knowledge of the design process and all goals for the product (e.g., is the device reusable?) My knowledge of the design process now includes exploring multiple solutions, refining the design, obtaining feedback, applying the feedback, and prototyping.”

“Knowledge of the design process and going through all the steps. I never realized how complicated it is, especially with the IRB process and validation testing. I will have a good idea of this now. Also, I feel like I could give my ideas to friends who are BioE's.”

## Discussion

Bioengineering and nursing students share a common curricular emphasis on practice and application in their undergraduate programs. Each group can support the other in their respective practice. For example, nursing students’ broad exposure to the clinic, clinicians, and patients positions them as valuable resources for bioengineering students aiming to design practical medical devices. Likewise, nursing students can gain valuable knowhow and experience in the design, development, testing, and regulation of medical products by collaborating with bioengineering students. This prepares nursing students to become active participants in user-centered problem solving and design as well as support activities such as human clinical testing. The data collected in this study make a strong case for such collaborations, with the outcomes demonstrating overwhelming benefits of the collaborative experience for both groups of students. A summary of the specific outcomes is provided for each research question.

RQ1: When developing a medical product, what effect on design self-efficacy is associated with an engineering-nursing student collaboration for the development of a medical product?

Significant effects for the bioengineering students relative to their confidence and success expectancy were associated with the interprofessional design experience. These effects included engineering design (ED) overall as well as the various steps of the engineering design process (EDP). Medium effect sizes for confidence (*d* = 0.70 each) and small effect sizes for success expectancy (*d* = 0.42 and *d* = 0.40) were found. Similarly, nursing students also experienced significant effects for confidence, with a large effect size (*g* = 1.19) for ED and a medium effect size for the EDP (*g* = 0.71). Thus, such interprofessional collaborations may be associated with significant effects for design confidence and success expectancy. The effect sizes for confidence were larger for the nursing students, suggesting a particularly impactful experience for them.

RQ2: When developing a medical product, what effect on interprofessional collaborative competencies attainment is associated with an engineering-nursing student collaboration

The retrospective pre-post differences from the interprofessional collaborative competency attainment survey (ICCAS) showed significant improvement for all 20 collaborative abilities for both the bioengineering and nursing students (*p* < 0.001). In fact, 19 of the 20 effect sizes were large for the nursing students, and 9 of 20 were large for the bioengineering students. Thus, interprofessional collaborations between bioengineering and nursing students may be associated with large, significant effects for collaborative competencies attainment. Additionally, these results suggest that such experiences may be particularly impactful for nursing students. Furthermore, a higher percentage of nursing students compared to bioengineering students reported overall improvement in interprofessional collaboration (83% vs. 68%), with 64% of nursing students reporting “much” better compared to 34% of bioengineering students. These latter results additionally support the possibility of a particularly impactful experience for the nursing students.

RQ3: When developing a medical product, what are students’ perspectives on an engineering-nursing student collaboration

During the interviews, the bioengineering students consistently valued the nursing students’ clinical/medical experience, knowledge, perspectives, ideas for unmet needs, and feedback (84% of interviewees), followed by access to clinical settings and contacts (46%). Similar advantages were stated by the bioengineering students when asked about the support they received in discovering unmet needs—the value of the nurse’s clinical experience, knowledge, and perspectives (64% of interviewees), the nurse as an idea feedback source (42%), the nurse as the project originator (27%), access to clinical settings (27%), and access to clinical contacts or end users (25%). The nursing students also supported quick feedback from clinical customers or stakeholders by facilitating connections for this feedback (55% of interviewees) and/or directly providing the feedback themselves (43%). Thus, the recurrent themes uncovered throughout the interviews aligned with the goals of this NIH education project, including providing clinical insights and perspectives to the product design process, supporting engineering development work in the clinical setting, and breaking down barriers for innovative engineering design for enhanced patient care.

During the interviews, the nursing students likewise realized the benefit they brought to the design team in terms of their clinical/medical experience, knowledge, perspectives, ideas for unmet needs, and feedback (59% of interviewees). They frequently highlighted the value of working as part of a team and establishing working relationships with the engineering students (71%). The nursing students also valued being *part* of the solution or solution process, having gained knowhow to do so (47% of interviewees), which reinforces the teamwork theme. The nurses believed they could be more innovative with interprofessional teamwork (56% of interviewees). Interestingly, the teamwork themes in the interview responses coincided with the largest effect sizes observed in the nursing students’ ICCAS results. The three largest effects were associated with the ability to understand the abilities and contributions of IP team members, the ability to provide constructive feedback to IP team members, and the ability to seek out IP team members to address issues. The largest effects from the ICCAS highlight the potential impact on nursing students’ abilities to create direct, constructive interactions with bioengineering students and understand what engineers bring to the team.

The nursing students also reported gaining knowledge about medical devices and the design process to support future innovativeness, which was a specific program goal. They gained knowhow on the solution process and associated their ability to be more innovative simply by recognizing unmet needs in the clinic (56% of interviewees). The nurses identified specific knowledge and skills they gained from their fellow engineering students—knowledge of engineered products, the medical product design process, sketching skills, and interprofessional communications, which will enhance their capacity for innovation in their careers.

A limitation of this research is the relatively modest response rates by the students to our survey instruments. The response rates were 22% for the bioengineering students and 21% for the nursing students. Future studies should entail student incentivization by way of extra course credit for survey completion. This study also did not consider direct assessment results such as a pre- and post-design challenge whereby student competencies could be directly assessed. This is also an opportunity area for future replication studies.

Implementation challenges that other educators should be aware of include the differing schedules of the bioengineering and nursing students. This typically complicated nursing students’ attendance at the weekly capstone class sessions since they were in the clinic at the time. This limited their team interactions with the bioengineering students as well as the engineering content they received. In addition, such partnerships involve administration and programmatic elements and issues. Given the budget restrictions associated with the NIH grant, the administration as well as content delivery was conducted by the capstone instructor. These issues potentially limited the full range of possibilities for the partnership.

## Conclusions

This research has demonstrated that nursing students can be valuable members of an interprofessional bioengineering design team focused on identifying and developing solutions for unmet clinical needs to ultimately improve care. The nursing student is a source of clinical knowledge and experience that can be leveraged for product design. The nursing student is also a potent facilitator of access to and contacts within the clinic. Further, educating nursing students in the basics of medical product development may impact creative opportunities in health care delivery. This research has demonstrated that bioengineering-nursing interprofessional educational experiences may be beneficial for design self-confidence and interprofessional collaborative competencies for both student groups. Given these and other benefits, such partnerships should be considered by schools of engineering and nursing for similar implementation. Further, the particularly impactful results for the nursing students suggest the desirability of nursing students becoming involved in bioengineering design partnerships, and this conclusion may extend to other health sciences students as well. This could be potentially transformative for the health care community at large.

## Data Availability

Due to IRB agreement, participant data are unable to be shared.
